# Systematic review of postoperative rehabilitation interventions after cranial cruciate ligament surgery in dogs

**DOI:** 10.1111/vsu.13755

**Published:** 2022-01-12

**Authors:** Leilani X. Alvarez, Jennifer A. Repac, Kristin Kirkby Shaw, Nashua Compton

**Affiliations:** ^1^ The Animal Medical Center New York New York USA; ^2^ College of Veterinary Medicine University of Florida Gainesville Florida USA; ^3^ Sound Veterinary Rehabilitation Center Shoreline Washington USA; ^4^ Veterinary Specialty Center of Seattle Lynnwood Washington USA

## Abstract

**Objective:**

To critically evaluate the evidence for rehabilitation interventions following surgery for cranial cruciate ligament disease (CCLD) in dogs.

**Study design:**

Systematic review.

**Methods:**

Google Scholar and Pubmed databases were searched for studies evaluating postoperative CCLD rehabilitation interventions from 1990 until March 2020 per the international Prospective Register of Systematic Reviews (PROSPERO). Each study was assigned a level of evidence score from I to IV and a risk of bias (RoB) score by 2 reviewers, and by a third reviewer, when consensus was not reached.

**Results:**

Nineteen studies met the inclusion criteria. Twelve comprised randomized, controlled trials (Level II), 6 were nonrandomized or nonblinded (Level III), and one was retrospective (Level IV). Nine studies had high RoB scores. Sixteen studies yielded positive results. Therapeutic exercise had the most studies with positive results but all had high RoB. Cold compression therapy had 3 supporting studies (2 Level II, low RoB). Extracorporeal shockwave yielded 2 positive Level II studies (low‐moderate and high‐moderate RoB) and photobiomodulation had 1 positive study (Level II, low RoB) with objective outcomes. A negative outcome was noted in 1 photobiomodulation study. There was 1 supporting study on electrical stimulation and there was none on low‐intensity pulsed ultrasound.

**Conclusion:**

This systematic review supports the use of rehabilitation interventions in recovery of postoperative CCLD in dogs; however, many studies had a high risk of bias.

**Clinical significance:**

There is a lack of class I level evidence in veterinary rehabilitation. This study supports therapeutic exercise and cold compression therapy for postoperative CCLD rehabilitation. Existing studies on other modalities are limited and demonstrate conflicting results.

## 1 INTRODUCTION

Cranial cruciate ligament disease (CCLD) is the leading cause of hindlimb lameness and stifle osteoarthritis in dogs.^1^, ^2^ Tibial plateau leveling osteotomy (TPLO), tibial tuberosity advancement (TTA), and extracapsular techniques are the most common surgeries performed for CCLD.^3^, ^4^ While systematic reviews have evaluated which surgical technique yields the best outcomes,^4^ no review has evaluated the effects of postoperative rehabilitation. Levels of evidence scales (Table 1) have been developed to review orthopedic surgical interventions^5^, ^6^ as well as nonsurgical treatments.^7^ Postoperative rehabilitation for CCLD is among the most common reasons for veterinary rehabilitation referral,^8^ yet, to the author's knowledge, there has been no published level I, systematic review evidence evaluating rehabilitation interventions in dogs after surgery for CCLD.

**TABLE 1:** Scale used to grade level of evidence^5^, ^7^

Level of evidence Class	Study Design	Examples/Comments
I	Evidence derived from multiple, randomized, blinded, placebo‐controlled trials in the target species	Systematic review (eg meta‐analyses)
II	Evidence derived from high quality clinical trials using historical controls	Randomized‐controlled clinical studies on naturally occurring disease in animals
III	Evidence derived from uncontrolled case series	Nonrandomized, prospective case comparison studies
IV	Evidence derived from expert opinion, and/or extrapolated from research or physiologic studies	Retrospective case comparison; laboratory studies

In human medicine, systematic reviews led to the formation of rehabilitation guidelines for physical therapy of patients recovering from anterior cruciate ligament reconstruction.^9^ Currently, an estimated 70‐71% of veterinary practitioners refer patients for postoperative orthopedic rehabilitation,^8^, ^10^ yet there is a lack of clear guidance on which modalities offer the greatest (if any) benefits. Modalities such as laser therapy (also known as photobiomodulation) and hydrotherapy are commonly considered as part of rehabilitation; however, other modalities such as custom exercise programs can also be utilized. This study may impact the number of practitioners and surgeons recommending rehabilitation and help guide the types of rehabilitation interventions that should be employed by practitioners and physiotherapists.

The objective of this study is to review systematically the literature for qualitative evidence that evaluates the effects of postoperative rehabilitation interventions in dogs recovering from CCLD surgery, evaluating levels of evidence^6^ and risk of bias according to systematic review guidelines.^11^, ^12^

## 2 MATERIALS AND METHODS

In accordance with the Preferred Reporting Items for Systematic Reviews and Meta‐analysis (PRISMA) guidelines,^11^ a systematic review was registered with the international Prospective Register of Systematic Reviews (PROSPERO), registration number CRD42019147702 (www.crd.york.ac.uk/prospero/). To complete PROSPERO registration, the study proposal undergoes a rigorous peer review process.

### 2.1 Search methods

Advanced searches (Table A1) were run on Google Scholar and Pubmed for the terms “cranial cruciate ligament” AND “canine” AND (“tibial plateau leveling osteotomy” OR “postoperative”) AND (“rehabilitation” OR “physiotherapy”).

Inclusion criteria consisted of studies in dogs with CCLD that had undergone surgery and received postoperative rehabilitation interventions with objective outcome measures. Retrospective, case‐control, and prospective studies dating from 1990 until March 2020 were considered for inclusion. There were no specifications for the length or frequency of the rehabilitation interventions.

Exclusion criteria included species other than dogs, lack of or inadequate description of rehabilitation therapeutic intervention, lack of control group, scientific abstracts, reviews, book chapters, expert opinion, case series, editorials, conference proceedings, publications earlier than 1990, absence of English translation, outcomes irrelevant to hindlimb or functional improvement, absence of surgical intervention, absence of control group activity level, pharmacologic studies evaluating only drug therapy and biologic interventions.

#### 2.1.1 Outcomes and prioritization

Primary outcomes were objective gait analysis – Total Pressure Index/Vertical Impulse/ Peak Vertical Force/Ground Reaction Forces (GRF) – and subjective lameness scores (graded on a scale out of 4 or 5). Radiographic osteotomy healing and pain scores (Canine Brief Pain Inventory and Canine Orthopedic Index) were considered secondary outcomes. Additional objective outcomes included range of motion (ROM) of hindlimb joints, thigh circumference, radiographic evidence of postoperative osteoarthritis with or without rehabilitation intervention, subjective functional tests (eg ability to rise), and owner outcome measures.

#### 2.1.2 Categorization of studies

Studies were categorized based on the primary therapeutic intervention investigated: exercise (including swimming, underwater treadmill therapy, land‐based exercise), cold compression therapy (CCT), extracorporeal shockwave therapy (ESWT), photobiomodulation (PBM), low‐intensity pulsed ultrasound (LIPUS), and electrical muscle stimulation (EMS).

#### 2.1.3 Level of evidence assessment

Level of evidence (LoE) was assigned as previously described^5^, ^6^, ^7^ to studies meeting inclusion criteria. Levels I‐IV were assigned by LXA and NC. When there was lack of agreement on LoE, a third reviewer, KKS, was enlisted to make the final determination.

#### 2.1.4 Risk of bias (RoB) assessment

Risk of bias (RoB) was assessed using the SYstematic Review Center for Laboratory animal Experimentation (SYRCLE) RoB tool adapted for clinical studies to assess methodology of scientific studies using animal interventions.^12^ Based on the responses to the adapted RoB questions (Table A2), LXA and JAR decided each study's RoB. A third reviewer, KKS, was used when RoB scores did not agree.

## 3 RESULTS

**FIGURE 1:**
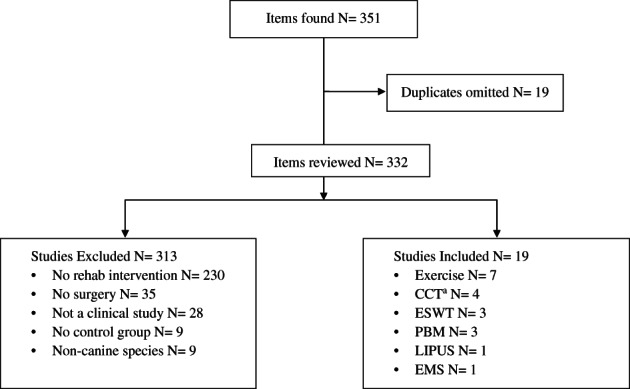
Flow chart showing the selection of studies evaluating the effect of rehabilitation interventions on postoperative cruciate ligament surgery in dogs

The search criteria resulted in 351 papers, of which 19 papers were omitted due to duplication (Figure 1). Of the 332 reviewed papers, 313 studies were excluded for not meeting inclusion criteria, with the majority of exclusions (N = 230) due to lack of rehabilitation interventions (Figure 1). Nineteen papers met the study criteria. Overall, 12 of 19 studies were high‐quality clinical trials (LoE II) and 16 yielded positive results (Tables 2 and 3). Six studies were nonrandomized (LoE III), and one retrospective study (LoE IV). Risk of bias (RoB) scores were assigned as “high” for 9, “high‐moderate” for 2, “low‐moderate” for 1, and “low” for 7. In total, there were 7 studies with LoE II with low RoB (Tables 2 and 3). Of these, 4 had positive outcomes,^21^, ^23^, ^24^, ^27^ 2 had negative outcomes,^29^, ^30^ and 1 had improvement in only 1 subjective outcome.^28^

**TABLE 2:** Summary of studies evaluating effect of exercise, diet, and cold compression therapy (CCT)

Intervention	Author	Year	Surgery	Study Design	N	Results	LoE^a^	RoB
Exercise	Marsolais^13^	2002	EC	Nonblinded prospective study	51	Rehab increased PVF and VI with no difference between contralateral limbs	III	High
	Marsolais^14^	2003	EC	Nonblinded prospective clinical study	20	Swimming increased stifle and tarsal range of motion compared to walking	III	High
	Monk^15^	2006	TPLO	Nonblinded prospective study	8	Rehab increased thigh girth and range of motion; no differences in lameness, weight bearing	III	High
	Jerre^16^	2009	EC	Nonblinded prospective study	39	Rehab did not improve lameness scores, thigh circumference, and visual analogue scores	III	High
	Romano^17^	2015	TPLO	Retrospective cohort study	236^b^	Rehab associated with improved functional scores and less unacceptable outcomes	IV	High
Exercise/diet	Verpaalen^18^	2018	TPLO	Double blind, randomized, clinical trial	48	Joint diet and rehab groups had lower radiographic OA scores; joint diet decreased PGE2 and delayed osteotomy healing; no differences in IL‐1B	II	High
	Baltzer^19^	2018	TPLO	Unblinded, randomized, clinical trial	48	Joint diet increased PVF and VI, rehab increased PVF and activity level; rehab and joint diet lowered pain and lameness scores	II	High
CCT	Rexing^20^	2010	EC	Nonrandomized, nonblinded placebo‐controlled clinical trial	24	Cold compression reduced thigh circumference (swelling); no difference in effect with concurrent bandage with electrical stimulation	III	High
	Drygas^21^	2011	TPLO	Double blind, randomized, placebo‐controlled clinical trial	34	Compression cryotherapy improved lameness, Glasgow pain scale score, pain threshold score, visual analogue score, range of motion	II	Low
	Kieves^22^	2016	TPLO	Unblinded, randomized, placebo‐controlled clinical trial	21	Cold compression therapy regardless of bandaging had no significant impact on weight bearing on operated limb, range of motion, or thigh circumference	II	High
	Von Freeden^23^	2017	TPLO	Double blind, randomized, placebo‐controlled clinical trial	30	Cryotherapy improved lameness, range of motion, and pain scores	II	Low

^a^ Abbreviations: EC, extracapsular; LoE, level of evidence; N= sample size; RoB, risk of bias; TPLO, tibial plateau leveling osteotomy.

^b^ Note: N = number of questionnaires.

**TABLE 3:** Summary of studies evaluating extracorporeal shockwave therapy (ESWT), photobiomodulation (PBM), low‐intensity pulsed ultrasound (LIPUS), and electrical muscle stimulation (EMS)

Intervention	Author	Year	Surgery	Study Design	N	Results	LoE^a^	RoB
ESWT	Gallagher^24^	2012	TPLO	Double blind, randomized, placebo‐controlled clinical trial	30	Shockwave improved patellar ligament thickness; no difference in ultrasonographic appearance	II	Low
	Barnes^25^	2015	TTA	Double blind, randomized, placebo‐controlled clinical trial	40	Shockwave increased densitometry at 4 weeks, but at 8 weeks there was no difference	II	Low‐Moderate
	Barnes^26^	2019	TPLO	Unblinded, randomized, placebo‐controlled clinical trial	16	Shockwave increased PVF; no differences in range of motion, thigh girth, pain score	II	High‐Moderate
PBM	Rogatko^27^	2017	TPLO	Double blind, randomized, placebo‐controlled clinical trial	27	Preoperative laser increased PVF; no change in lameness, response to manipulation	II	Low
	Renwick^28^	2018	TPLO	Double blind, randomized, placebo‐controlled clinical trial	95	Gait canine orthopedic index improved in laser group; no change in osteotomy healing, wound healing, time to cessation of NSAIDs	II	Low
	Kennedy^29^	2018	TPLO	Double blind, randomized, placebo‐ controlled trial	12	Laser had no impact on ground reaction forces, pain scores, synovial fluid, or radiographic changes	II	Low
LIPUS	Kieves^30^	2018	TPLO	Double blind, randomized, placebo‐controlled clinical trial	50	Low‐intensity pulsed ultrasound had no impact on TPI or bone healing	II	Low
EMS	Johnson^31^	1997	EC	Unblinded, randomized, placebo‐controlled clinical trial	12	Electrical stimulation improved lameness scores, thigh circumference, and decreased radiographic osteoarthritis; did not impact ground reaction forces	III	High‐moderate

a Abbreviations: EC, extracapsular; N= sample size; LoE, level of evidence; RoB, risk of bias; TPLO, tibial plateau leveling osteotomy; TTA, tibial tuberosity advancement.

### 3.1 Exercise

Marsolais et al. performed a prospective controlled clinical trial in dogs comparing the effects of rehabilitation (n = 25) versus activity restriction (n = 26) after extracapsular repair.^13^ Rehabilitation included swimming, passive ROM (PROM), therapeutic walks, and massage during the third, fifth, and seventh postoperative weeks. Aquatic sessions consisted of alternating 1 minute intervals of swimming and rest, totaling 5‐10 min of active swimming time. Peak vertical force (PVF) and vertical impulse (VI) were greater at 6 months postoperatively in dogs that received rehabilitation. Lack of randomization and blinding of investigators resulted in level III evidence and high RoB. Further, “therapeutic walks” were not adequately described to enable study replication.

Marsolais et al. investigated kinematics of swimming and walking in dogs recovering from extracapsular repair (n = 7) compared with healthy dogs (n = 13).^14^ The swimming protocol was the same as in the preceding study,^13^ except all dogs swam at least 10 min total per day based on individual fitness. Both groups demonstrated increased tarsal and stifle flexion, and subsequently range of motion, during swimming compared to walking. This study was deemed level III with high RoB as it lacked blinding.

Monk et al. investigated the effects of rehabilitation compared to a home exercise plan in dogs recovering from TPLO.^15^ Treatments included PROM, massage, underwater treadmill, and functional weight‐bearing exercises, 3 times per week, for 6 weeks. This study also lacked blinding and randomization of groups and was thus classified as level III evidence with high RoB. Given the small sample size (n = 8), type II error must also be considered. At 6 weeks, thigh circumference and stifle ROM were larger in the rehabilitation group (n = 4) compared with the home exercise group (n = 4). Weight‐bearing and lameness scores were not different between groups.

Jerre et al. compared the effects of home exercise (n = 19) versus swimming and electrical stimulation therapy (n = 20) in 39 dogs post extracapsular surgery.^16^ There were no differences in owner assessment, lameness score, and stifle stability between the two groups. The focus of this study was on outcomes of a modified extracapsular technique rather than the effects of rehabilitation. This study was classified level III evidence with high RoB due to lack of statistical analysis and blinding of assessors.

Romano et al. analyzed 236 veterinary questionnaires regarding recovery rates of dogs receiving rehabilitation following TPLO.^17^ As a retrospective cohort study, this was level IV evidence with high RoB. Dogs receiving rehabilitation were more likely to achieve functional outcomes at 8 weeks.

Verpaalen et al. and Baltzer et al. published 2 studies on the same patient population (n = 48) investigating the effects of rehabilitation and omega‐3 and protein‐enriched diet in dogs after TPLO.^18^, ^19^ Twelve dogs were enrolled in each group (rehabilitation only, diet with rehabilitation, diet only, control). Rehabilitation consisted of 2 weeks of home exercises and formal in‐hospital rehabilitation at weeks 4‐8. Synovial fluid analysis, radiographic assessment, GRF, accelerometry, pain scores, and limb girth were measured. These studies are considered level II evidence but they had high RoB as investigators and caregivers were not blinded to rehabilitation treatments. The study was also sponsored by the manufacturers of the therapeutic diet. Rehabilitation only treated dogs showed greater PVF, lower pain and lameness scores, increased activity levels, and decreased progression of osteoarthritis. Dogs fed supplemented diets, with or without rehabilitation, had higher PVF, VI, lower levels of PGE2 and slower time to complete osteotomy healing.

### 3.2 Cold compression therapy (CCT)

Rexing et al. examined the effects of cold compression therapy, microcurrent electrical therapy, and bandaging applied after extracapsular repair in dogs.^20^ There were five dogs each in the bandage, bandage with CCT, and CCT only groups. Cold compression consisted of a large cold pack (stored at 30 °F) wrapped with reusable elastic bandages from the stifle to the tarsus and held in place for 20 min. Six dogs received microcurrent electrical therapy only. This study was considered level III; however, it had high RoB due to unblinded observers. CCT (with or without bandaging) and microcurrent electrical therapy with a bandage‐reduced limb circumference after 74 h.

Drygas et al. evaluated the effect of postoperative CCT (n = 17 treatment, n = 17 control) in dogs recovering from TPLO.^21^ Cold compression therapy was performed every 6 h for 30 min for four total treatments using a Game Ready Equine unit (Cool Systems, Berkeley, California). This randomized, double‐blinded controlled clinical trial was considered level II evidence with low RoB. This study supported short‐term benefits of CCT. Dogs receiving CCT had lower visual analogue and Glasgow pain scale scores, lower pain threshold scores, decreased stifle circumference, and increased ROM in the first 24 h postoperatively. No benefit was detected after 14 days.

Kieves et al. compared CCT, bandaging, and CCT with bandaging in dogs following TPLO (n = 7 per group).^22^ CCT dogs had 20‐minute treatments every 4 h for the first 24 h, then every 6 h for the remaining 24 h using a canine Game Ready. Investigators measured standing weight bearing of the surgical limb, stifle ROM, and limb girth. No differences were observed between groups at 12, 24, and 36 h. A post hoc power analysis showed 321 subjects were required to observe a difference between groups, suggesting type II error. While this study fit level II evidence criteria, it was classified as high RoB due to unblinding and small sample size.

Von Freeden et al. compared the effects of two CCT protocols (cold compression device, pump model no. PPRT‐01, and wrap and gel pack model no. LCSW‐12, Lite‐Cure, Companion Animal Health, Newark, Delaware) in dogs undergoing TPLO.^23^ Thirty stifles from 27 dogs were randomly allocated into 3 groups (n = 10/group). Group I received CCT once preoperatively and postoperatively; group II received 4 postoperative treatments 6 h apart, and group III received no CCT. The CCT dogs had greater stifle ROM and lower Glasgow pain scores 24 h after surgery. After 10 days, both CCT groups had lower lameness scores compared with the control group. Forty‐two days after surgery, ROM was greater in both CCT groups compared to the control group. There were no differences in outcomes between the CCT groups at any time point. This was a double‐blinded, placebo‐controlled trial contributing Level II evidence with a low RoB. However, 3 patients had bilateral TPLO constituting a unit of analysis error. It is unknown how treatment or condition of one limb affects outcomes in the contralateral limb and it was unclear if limbs were individually randomized.

### 3.3 Extracorporeal shockwave therapy (ESWT)

Gallagher et al. evaluated the effects of ESWT on patellar ligament desmitis in dogs recovering from TPLO.^24^ Extracorporeal shockwave therapy dogs (n = 19) received shockwave therapy at 4 and 6 weeks postsurgery and were compared with a control group (n = 11) that did not receive treatment. The ESWT delivered 600 pulses at energy level 6 (0.15 mJ/mm^2^) with a 5 mm trode focused on the patellar ligament using an electrohydraulic generator (PulseVet VersaTron, Alpharetta, GA). At 4, 6, and 8 weeks postoperatively, patellar ligament thickness was measured on radiographs and ultrasonographic ligament pathology was graded. This was a prospective, randomized, blinded, controlled clinical trial constituting level II evidence with low RoB. Mean patellar ligament thickness measured radiographically was found to be lower in the treatment group at 6 and 8 weeks compared to the control group. No difference was found in the ultrasound scores between treatment and control groups.

Barnes et al. assessed the effect of ESWT on osteotomy healing with autogenous cancellous bone grafting in dogs following tibial tuberosity advancement (TTA).^25^ Forty stifles from 39 dogs were randomized into four groups of 10 dogs: autologous bone graft with ESWT, autologous bone graft without ESWT, ESWT without autologous bone graft, and neither therapy. The ESWT groups were treated immediately postoperatively and at 4 weeks postsurgery. Extracorporeal shockwave therapy consisted of 1000 pulses at energy level 6 (0.15 mJ/mm^2^) delivered to the stifle with a 5 mm trode (PulseVet). Postoperative radiographic densitometry was measured at weeks 0, 4, and 8. This unblinded randomized, controlled study was considered level II evidence with a low‐moderate RoB. At 4 weeks, the ESWT with bone graft group demonstrated higher osteotomy gap density compared to both the no treatment and ESWT only groups. No significant difference was found between any of the groups at 8 weeks.

Barnes et al. investigated the effect of ESWT in 16 dogs after TPLO (n = 9 ESWT; n = 7 control).^26^ The ESWT consisted of 1000 pulses 0.15 mJ/mm^2^ delivered to 4 areas around the stifle with a 5 mm trode (PulseVet). Extracorporeal shockwave therapy was administered at 0 and 2 weeks and additional outcomes (pain score, thigh girth, goniometry) were included. As in the Barnes TTA study,^25^ no difference in osteotomy healing was found between ESWT and control groups at 8 weeks. However, the ESWT dogs had greater PVF and VI than control dogs. Thigh girth, pain scores, and goniometry were not different between cohorts. The pain score raters were unaware of group assignment; however, blinding of individuals measuring goniometry and thigh girth was omitted. This study was therefore considered level II with a high‐moderate RoB.

### 3.4 Photobiomodulation

Rogatko et al. evaluated effects of PBM on 27 dogs recovering from TPLO (n = 12 PBM and n = 15 control).^27^ The GRF, radiographic bone healing, and assessment of lameness, behavior, movement, and response to manipulation were noted. Photobiomodulation (PBM) (800‐900 nm dual wavelength, 6 W, 3.5 J/cm2, 100 cm^2^ area) with a class IV laser (K‐series1200: K‐Laser, Franklin, Tennessee) or sham treatment was administered preoperatively to the stifle. The PVF was greater in the PBM than the control at 8 weeks. The age of dogs in the PBM group (6.6 ± 1.6 years) was greater than that of the control group (4 ± 2 years). No other significant differences in other parameters were found.

Renwick et al. performed a PBM study on dogs undergoing TPLO with a preoperative and postoperative treatment protocol applied to the proximal tibia and lumbosacral area using a class IV laser (class IV K laser; 660 nm red at 800, 905 and 970 nm infrared, maximum 15 W continuous wave, 20 W peak pulsed wave).^28^ Ninety‐five dogs were included in the study (n = 51 PBM and n = 44 control). The outcomes measured included the adjusted Canine Orthopedic Index (ACOI), Liverpool Osteoarthritis in Dogs, radiographic osteotomy healing, wound healing, and duration of nonsteroidal inflammatory drug administration. Dogs in the PBM were shown to have improved subjective outcomes in ACOI gait. All remaining outcomes were not different between groups.

Kennedy et al. investigated the effects of PBM using a class II laser (3LT PL5000, Erchonia Lasers Ltd, Wallingford, England) preoperatively and postoperatively applied to the stifle and L6‐7 lumbar area in dogs after TPLO.^29^ Twelve dogs (6 PBM and n = 6 control) were enrolled. There were no significant differences in all outcomes measured (Canine Brief Pain Inventory, accelerometry, ground reaction forces, radiographic bone healing, soft tissue healing, and osteoarthritis (OA) scoring, lameness, Glasgow Composite Pain Scale, synovial fluid analysis). Interestingly, control dogs demonstrated lower owner pain scores at weeks 1‐5 and higher GRF until week 8.

### 3.5 Low‐intensity pulsed ultrasound therapy (LIPUS)

Kieves et al. conducted a study on the effects of LIPUS on radiographic healing and limb function in 50 dogs after TPLO (n = 25 each LIPUS and control).^30^ There were no significant differences in radiographic scores and objective gait analysis using a pressure sensing walkway between the LIPUS and sham group. This study was a double‐blinded, placebo‐controlled investigation providing level II evidence with low RoB. The LIPUS protocol was described in detail (1.5 MHZ pulsed at 1 kHZ with 20% duty cycle at 30 mW/cm2 intensity for 20 min) and the study was free of design‐specific risks of bias.

### 3.6 Electrical muscle stimulation

Johnson et al. studied the effect of electrical muscle stimulation (EMS) in dogs recovering from extracapsular repair 3 weeks after experimentally transecting the cranial cruciate ligament.^31^ Twelve dogs were in this study (n = 6 EMS and n = 6 control dogs). The EMS dogs received 30 minute treatments once daily, 5 days per week, for 4 weeks. Dogs with EMS had larger limb circumference, decreased radiographic OA scores, and lower lameness scores compared to the control group. There were no differences in GRF and stifle ROM between groups. This randomized, controlled clinical trial constituted level III evidence with high‐moderate RoB. The orthopedist performing the visual lameness score was unblinded to patient group and the frequency and pulse width EMS settings were omitted. Study subjects did not have naturally occurring disease.

## 4 DISCUSSION

This systematic review of rehabilitation interventions demonstrated the highest number of studies in support of exercise‐based therapy for dogs recovering from CCLD surgery; however, most studies had high risk of bias. The studies constituting the highest level of evidence (LoE II) and lowest risk of bias showed conflicting results for efficacy. Cold compression therapy (CCT) was the only modality to have 2 supporting Level II studies with low RoB. The lack of quality evidence supporting other modalities, such as photobiomodulation and electrical muscle stimulation, may be disproportionate to their prevalence in the field of rehabilitation.

Formal exercise‐based rehabilitation programs in dogs post CCLD surgery is supported by 6 of the 7 exercise studies in this review.^13^, ^14^, ^15^, ^17^, ^18^, ^19^ However, more studies with high LoE and low RoB are needed. In the studies lacking randomization, selection bias may affect outcomes of animals receiving rehabilitation. Owners subscribing to rehabilitation may be more diligent in other aspects of care (eg activity control, weight management, administration of medications). Unblinding of investigators may be less important in studies collecting objective outcome measures (such as GRF and kinematics) compared with those collecting subjective data (such as Client Specific Outcome Measures and lameness scoring). It is also challenging to keep rehabilitation treatment groups blinded given they are receiving exercise interventions that are difficult to simulate in a sham group compared to modality interventions. It should be noted that some studies included in the category of therapeutic exercise included manual modalities (such as massage and PROM)^15^ that would not traditionally be considered “exercise”; however, there were no studies that evaluated only manual therapies. Overall, exercise interventions showed benefits, but there is a wide range of exercise techniques and further research is indicated to explore the optimal exercise protocol for postoperative CCLD.

Given that, in humans, exercise therapy is the mainstay of evidence‐based rehabilitation therapy for management of anterior cruciate ligament rupture,^9^, ^32^ further investigation should be pursued in veterinary medicine that follows the guidelines set out in the fundamental principles of rehabilitation,^33^ including gradual progression of neuromuscular retraining, as well as dynamic functional tasks. This could lead to development of standardized guidelines in canine postoperative CCLD rehabilitation, similar to the Multicenter Orthopaedic Outcomes Network (MOON) guidelines in humans.^9^

Existing evidence from 3 studies support the use of CCT in dogs recovering from CCLD surgery (Table 2). However, conclusions drawn in one of these studies without observer blinding should be cautiously interpreted.^20^ Thigh girth has also been shown to have high intraobserver and interobserver variability.^34^ Results from the Von Freeden et al. study^23^ found increased ROM in both CCT groups 42 days after surgery. This bears contrast with the study by Drygas et al.,^21^ which did not find differences between groups 14 days after surgery. This may be due to the different types of CCT devices that were used in each study. Overall, CCT was the only modality demonstrating positive results in 2 studies with a high level of evidence and low RoB.

All studies on ESWT were well conducted, demonstrated positive results, and were considered to be level II evidence; however, only one study had low RoB.^24^ There may be a positive effect of ESWT on early bone healing,^25^ but this is likely transient.^25^, ^26^ Radiographic improvement of patellar desmitis was demonstrated,^24^ but the clinical significance of this is unknown. The ESWT showed no long‐term effect on bone healing; however, osteotomy healing appears to be independent of functional outcomes,^17^, ^26^ and thus may not be a clinically relevant outcome measure. All ESWT studies utilized electrohydraulic generators. There is currently no evidence supporting other types of ESWT (electromagnetic, piezoelectric, or radial) for postoperative CCLD in dogs.

All 3 PBM studies were well constructed, double‐blinded, placebo controlled clinical trials and considered Level II evidence with low RoB.^27^, ^28^, ^29^ However, existing veterinary literature on PBM yields mixed conclusions. Only one study showed improvement in objective outcome measures.^27^ Adding to the confusion, studies vary in laser power and wavelength, protocols, and dosage units. This creates a challenge in interpreting and comparing outcomes. While research suggests class IV lasers may provide some benefits,^27^, ^28^ further research is warranted. Notably, the single study that used a class II laser,^29^ yielded no benefit of PBM. Practitioners are cautioned in the use of PBM in postoperative CCLD given the current lack of evidence.

The level of evidence is low for both low‐intensity pulsed ultrasound and electrical muscle stimulation in dogs. The use of these modalities for postoperative CCLD in dogs is not supported by this review.

### 4.1 Limitations

This review has several limitations. Search terms entered for advanced search criteria were designed to be inclusive of all methods used to surgically treat CCLD by including “OR” terms under “postoperative” and inclusive of all rehabilitation interventions by including “OR” terms under “rehabilitation”; however, the search methods may have inadvertently excluded relevant studies. Despite abiding by PRISMA guidelines to assess RoB and LoE objectively, subjectivity remains in these scoring systems. This study implemented higher criteria than most veterinary systematic reviews by including RoB as part of the evaluation criteria. Studies that were identified as high RoB, should not be interpreted as low‐quality evidence. The RoB assessment adds a higher level of criteria that readers can use to help guide them in interpreting the data. This systematic review accounted for both LoE and RoB.

The wide variety in outcome measures prohibited a meta‐analysis of the data, thereby resulting in less rigorous evaluation of claims and clinical recommendations. The rehabilitation interventions evaluated also include a wide variety of modalities and treatment periods, which cannot be conflated to have equal efficacy. Differences in outcome between various studies may have been related to different surgical techniques, undetected meniscal injury, persistent instability, lack of ongoing pain management to address osteoarthritis, or other variables not related to rehabilitation interventions.

### 4.2 CONCLUSIONS

This study presents a systematic review to evaluate rehabilitation interventions in dogs recovering from CCLD surgery. The evidence reported here supports exercise‐based therapy and cold compression therapy for postoperative CCLD rehabilitation; however, more rigorous prospective clinical trials are needed with low risk of bias before specific clinical recommendations can be made. Practitioners and surgeons are cautioned against using modalities in postoperative CCLD recovery that currently lack clinical evidence.

## CONFLICT OF INTEREST

The authors declare no conflicts of interest related to this report.

## Supporting information


**TABLE A1**. Search strategy with search terms and yield

**TABLE A2**. Adapted risk of bias (RoB) tool used for this study^12^
