# Supplementary material for: Systematic review of postoperative rehabilitation interventions after cranial cruciate ligament surgery in dogs
Source: Vet Surg. 2022 Jan 12;51(2):233–43. doi: 10.1111/vsu.13755 (PMC9303706; doi:10.1111/vsu.13755)
Supplement: Supplementary file 1 — TABLE A1. Search strategy with search terms and yield [file VSU-51-233-s002.docx]

**APPENDIX A**

TABLE A1. Search strategy with search terms and yield

| **Search Terms** | |
| --- | --- |
| ((("canine s"[All Fields] OR "dogs"[MeSH Terms] OR "dogs"[All Fields] OR "canine"[All Fields] OR "canines"[All Fields]) AND ("rehabilitation"[All Fields] OR "physiotherapy"[All Fields]) AND "postoperative"[All Fields]) OR "tibial plateau leveling osteotomy"[All Fields]) AND 1990/01/01:2020/03/01[Date - Publication] AND "english"[Language] | |
| Search Yield = 351 | |
| PubMed: 242 | Google Scholar: 109 |
