# Supplementary material for: Systematic review of postoperative rehabilitation interventions after cranial cruciate ligament surgery in dogs
Source: Vet Surg. 2022 Jan 12;51(2):233–43. doi: 10.1111/vsu.13755 (PMC9303706; doi:10.1111/vsu.13755)
Supplement: Supplementary file 2 — TABLE A2. Adapted risk of bias (RoB) tool used for this study 12 [file VSU-51-233-s001.docx]

**APPENDIX A**

TABLE A2. Adapted risk of bias (RoB) tool used for this study^12^

| **RoB Level** | **Criteria** |
| --- | --- |
|  |  |
| High | Lack of blinding of outcome assessor |
|  | Inadequate concealment of group allocation |
|  | Lack of blinding of caregivers and investigators |
|  | Unavailable study protocol |
|  | Not all expected outcomes available |
|  | Not free of influence of inappropriate funders |
|  | Not free of design-specific risks of bias (e.g. no stats on covariables) |
| Moderate | Partial blinding |
|  | Lack of balanced distribution of baseline characteristics in intervention and control groups |
| Low | Adequate allocation sequence generation and application (randomization) |
|  | Adequate adjustment for unequal baseline characteristics in analysis |
|  | Any missing outcome data is unlikely to be related to true outcome (e.g. technical failure) |
|  | Balanced numbers and similar reasons for missing outcome data across groups |
|  | Missing outcome data imputed using appropriate methods |
|  | Random selection of animals for outcome assessment |
|  | All animals included in analysis |
|  | No unit of analysis errors |
|  | Replacement of dropouts of control and experimental groups from original population |
